# Death from Somnoforme in Rockford, Ill

**Published:** 1906-10

**Authors:** 


### DEATH FROM SOMNOFORME IN ROCKFORD, ILL.

Mrs. Grace Herbig, wife of William Herbig, died yesterday afternoon in the operating room of the Hayes Dental parlors, West State street, following the extraction of a tooth.

Dr. T. T. Woollens, general manager of the offices in this city of Chicago, was the operator, administered the anaesthetic and drew the tooth. Finding his patient was not responding he used usual means to bring back consciousness and at the same time sent for physicians to aid him. Before any arrived Mrs. Herbig was dead.

The evidence of Dr. Woollens was the most important before the coroner's jury last night, his story being corroborated as far as their knowledge went by Dr. Stone and Miss May Hogan, 2523 West State street, the office attendant.

He related the facts in relation to the standing of his concern and exhibited his license from the state and its county recorder. He then related the events preceding and accompanying the death of Mrs. Herbig.

She entered the office and after waiting for another patient to have her work attended she expressed a desire to have a tooth drawn, as it had been troubling her. The dentist, as usual, tried to persuade her to have the offending molar treated and saved by filling, but she insisted on its removal and asked that somnoforme, the anaesthetic used by this firm, be used for the purpose of removing the pain.

This was applied and the tooth removed, a small portion of one of the roots remaining.

### WOMAN WANTED ANAESTHETIC.

Dr. Woollens suggested that this fragment might be removed with little pain, but Mrs. Herbig asked for a repetition of the anaesthetic as she had felt no pain under its use and desired it again. After waiting the usual time, about half an hour, to allow the effects of the first dose to disappear, the second was administered and the fragment removed.

Shortly after its application Doctor Woollens noticed that she was not reacting in the usual way and at once lowered the chair to place her head low, the common procedure. He then administered a small drink of whisky and this doing no good injected hypodermically nitro-glycerine and then whisky and finally tried artificial respiration.

He soon saw that his efforts were useless and instructed his office attendant to phone for a physician and sent the office boy on the same errand.

It was almost fifteen minutes before a physician could be secured and in the meantime Mrs. Herbig had passed away, never regaining consciousness.

The husband, William Herbig, was placed on the stand and stated that his wife had been a strong and healthy woman, never having been ill and never showing signs of heart trouble as far as he knew. The trouble with her teeth and the birth of her children were the only occasions when she had sought medical aid as long as he had known her, some thirteen years.

In view of the incompleteness of the evidence as to the actual cause of death the jury adjourned until this evening. In the meantime it is probable an autopsy will be held to determine, if possible, if any heart trouble can be demonstrated or if any other indication exists to throw light on the case.—*Rockford Daily News*, Sept. 13, 1906.

